# Common Neurologic Features of Lyme Disease That May Present to a Rheumatologist

**DOI:** 10.3390/pathogens12040576

**Published:** 2023-04-09

**Authors:** Swati Govil, Eugenio Capitle, Alexandra Lacqua, Reena Khianey, P.K. Coyle, Steven E. Schutzer

**Affiliations:** 1 Department of Medicine, Rutgers New Jersey Medical School, Allergy, Immunology, and Rheumatology, Newark, NJ 07103, USA; 2 Department of Medicine, Florida Atlantic University, Boca Raton, FL 33486, USA; 3 Department of Neurology, Renaissance School of Medicine, Stony Brook University, Stony Brook, NY 11794, USA

**Keywords:** Lyme disease, neurologic Lyme disease, rheumatology, *Borrelia burgdorferi*, tick-borne disease

## Abstract

Lyme disease, caused by *Borrelia burgdorferi* (Bb) infection, has a broad spectrum of clinical manifestations and severity. Patients with possible Lyme disease may seek out or be referred to rheumatologists. Today, the most common reason to engage a rheumatologist is due to complaints of arthralgia. After skin, neurologic manifestations of Lyme disease are now among the most common. Therefore, it is important for rheumatologists to be aware of clues that suggest neurologic Lyme disease and prompt help from a neurologist experienced with Lyme disease.

## 1. Introduction 

### 1.1. Purpose

Rheumatologists may see patients from Lyme disease-endemic areas who complain about joint pain or who are referred to them by another physician.

Today, Lyme disease with true arthritis occurs far less than cases with arthralgias and neurologic features. Neurologic manifestations are now recognized as among the most common extracutaneous manifestations. Recognition of suggestive neurologic features may warrant a referral to a neurologist who is familiar with Lyme disease.

The purpose of this paper is to alert the rheumatologist, particularly in the United States, of the common neurologic Lyme disease features which should trigger a referral.

### 1.2. Background

Lyme disease is a tick-borne illness caused by *Borrelia burgdorferi (Bb).* In 1977 “Lyme arthritis” was first recognized as neighborhood outbreak of what was believed to be idiopathic juvenile rheumatoid arthritis [1]. The fact that a systemic infectious disease caused these manifestations and that the etiologic agent was *B. burgdorferi*, transmitted through a tick bite [2,3] was not realized until later. In particular, the cases were recognized to be arthritis, especially of the knee. The belief that arthritis and arthralgias, primarily of the knee in endemic areas, may be due to Lyme disease has encouraged primary care providers and patients themselves to seek out a rheumatologist. Rheumatologists have played a major role in diagnosing and treating Lyme disease, and can be an informed gatekeeper when neurologic Lyme disease may be present. They can also be a source of well-characterized body fluid samples and specimens to help research on Lyme disease and other fields of medicine.

Lyme disease is highly seasonal in temperate climates. Approximately two-thirds of the cases from 1992 to 2006 have reported onset dates in June, July, or August. The seasonality of case occurrence varies geographically, with the beginning of the main transmission season occurring earlier in southern endemic states and later in the northern endemic states [1]. The increasing prevalence of Lyme disease further solidifies the importance of disease symptom and sign recognition [4]. Exposed patients may complain about pain (in joints, especially large joints, muscles, spine, and head), fatigue, and cognitive impairment. They may also exhibit skin lesions. However, because skin lesions related to Lyme disease occur at the onset of the disease, and the main symptoms of arthralgias occur later, it is not likely that the rheumatologist would see them when the patient first presents. Hence, a good history regarding this and exposure is important.

Gathering subjective and objective data is the first step in differentiating Lyme disease from other diseases, such as a viral syndrome. Immediate suspicion for Lyme disease should occur when there is potential tick exposure in an endemic area, and the subject has an expanding skin lesion known as erythema migrans (EM) [5,6,7,8,9,10]. The lesion often has varied appearances [9,10,11] (see below). Further information about symptoms, history of Lyme disease, and family members with similar symptoms and signs should be obtained. Awareness of suggestive joint/musculoskeletal (Table 1) and neurologic clues for Lyme disease are helpful (Table 2). However, due to the non-specific nature of these manifestations, accurate diagnosis will generally involve a laboratory investigation.

For a rheumatologist, a detailed history and comprehensive physical exam is pertinent to make a diagnosis of Lyme disease and rule out other disorders.

The most common neurologic features of early disseminated Lyme disease are shown in Table 2. Although the initial discovery of Lyme disease involved arthritis, this feature has now diminished markedly [16]. The manifestations are still frequently reported, but largely involve subjective joint pain (arthralgias) rather than true arthritis. This leads to an overestimation of Lyme arthritis incidences. In a recent Canadian study of 1230 patients reported to have Lyme disease, the overall incidence of arthritis was 0.028%. Early disseminated infections had 94% of neurologic complaints, while late disseminated infections had a 55% rate of neurologic complaints compared to 93% of arthralgias [16]. Of the 475 cases reported to have late-stage Lyme disease, only 35 (7.4%) manifested true arthritis, while 440 (92.6%) had arthralgias. Neurologic manifestations were noted in 259 (54.5%) cases [16]. Thus, common extracutaneous manifestations are now found to be neurologic.

The rheumatologist may be sought out directly by patients or referred by primary care physicians. Because the nervous system is among the most commonly involved body system other than the skin, the remainder of this article is oriented to the neurologic clues a rheumatologist is likely to encounter, especially in North American cases.

### 1.3. Elicitation of a History of Skin Lesions Resembling a Form of Erythema Migrans (EM)

Several studies with microbiologic proof of *B. burgdorferi* infection, demonstrated that the often described “classic” bull’s-eye lesion with central clearing occurred far less than “non-classic” atypical EM lesions [9,10]. In fact, the classic appearance occurs more frequently in southern tick-associated rash illness (STARI) than Lyme disease [17]. Mimics, such as a drug eruption, may appear as a classic lesion [6]. Photographs of the classic and varied appearances are shown on the CDC website [11].

Although it is unlikely that patients will be seen by a rheumatologist for an asymptomatic skin lesion, such as EM, it is worth seeking a possible occurrence in the patients’ history. EM can be present initially with or without symptoms. An EM lesion may not be noticed because it is usually painless and does not itch. Atypical EM appearances are observed in 25–30% of all cases, even in PCR-positive cases [10]. The EM lesion may occur 4–14 days after a tick bite [6]. The appearance of a rash within 24 h of a suspected tick bite supports a hypersensitivity reaction rather than EM. EM can be present as many various appearances. The recognition of EM is often dependent on the knowledge and experience of the clinician looking at the skin lesion, which now can be supported by adjunctive laboratory tests [18,19].

## 2. Neurologic Features of Lyme Disease

The more common neurologic features of early disseminated Lyme disease are shown in Table 2. These include focal weakness due to a cranial nerve VII palsy (rarely other cranial nerves are involved), aseptic meningitis syndrome, and acute painful radiculoneuritis. Very infrequent manifestations include cerebrovascular issues, including vasculitis, encephalomyelitis, and intracranial hypertension syndromes, in adolescents. Patients with these rare manifestations would likely present to neurologists directly.

Facial nerve palsy is a major manifestation of neurologic Lyme disease. It is important to determine if there is unilateral weakness (with inability to close the accompanying eye lid or wrinkle the forehead). One should ask about tearing, hearing, or taste abnormalities involved on the same side of the weakness and anterior portion of the tongue. These signs may be subtle but confirm a peripheral cranial nerve VII involvement. When there is bilateral facial nerve involvement in a patient with endemic area exposure, Lyme disease is among a differential diagnosis list. This list includes Guillain–Barré syndrome, HIV, sarcoidosis, Epstein–Barr virus infection, and lymphoma. Lyme disease can sometimes involve other cranial nerves (including III, IV, VI) to produce double vision.

Symptomatic lymphocytic/mononuclear meningitis due to Lyme disease is largely indistinguishable from viral meningitis, with headache, fever, photosensitivity, and stiff neck. In a patient with a clinical presentation suggesting acute meningitis, cerebrospinal fluid (CSF) examination is mandatory to guide diagnosis and therapy, including those due to other pathogens.

The third neurologic syndrome of early dissemination is acute painful radiculoneuritis. This is more commonly seen in Europe. The term “Garin-Bujadoux-Bannwarth syndrome” (or “Bannwarth syndrome”) has been applied to the constellation of painful radiculoneuritis (the hallmark of the syndrome, with severe spinal pain) with variable motor weakness, sometimes accompanied by facial nerve palsy. There is a robust CSF pleocytosis [20], despite absence of headache and meningeal signs. Fuller descriptions are published [20,21,22]. Spine pain (neck or mid/lower back pain along the spine) is typically prominent and may have radicular features, such as scapular winging and dermatomal sensory loss. Imaging will likely be unable to diagnose painful radiculoneuritis.

The clinical manifestations of late neurologic Lyme disease include subtle encephalopathy, rare encephalomyelitis (most cases are European), and possible neuropathies, such as mononeuropathy multiplex, or a subtle sensory axonal peripheral neuropathy. A mild chronic encephalopathy may be the most common neurologic manifestation in patients with late-stage Lyme disease. The symptoms tend to be diffused and nonspecific, and patients typically report memory loss, sleep disturbance, fatigue, and depression [23]. There are currently debates on whether this represents a central nervous system (CNS) infection, or a systemic mechanism.

## 3. Laboratory Tests as an Adjunct to Diagnosis of Lyme Disease Involving the Nervous System

It is certainly helpful if one can document characteristic involvement, such as EM. However, this may not be apparent. Laboratory tests can be useful to document exposure to *B. burgdoferi*. Currently, the only FDA-approved tests are antibody tests. These are indirect tests that measure the host humoral response to the pathogen. A single test cannot prove active infection, but rather exposure. Limitations and caveats have been discussed elsewhere [18,24]. However, it should be noted these types of laboratory tests are undergoing relatively fast change, so it is important to keep abreast of the field [18,24]. Currently, two types of two-tiered tests are approved. The older one is now designated as the standard two-tiered test (or two-step approach) (STTT). The first tier has commonly been an ELISA assay, and if positive or borderline, is followed by a second test. For many years, the second test has been a Western immunoblot. Visual interpretation of the blot is subjective and involves counting different protein “bands” thought to represent specific *B. burgdorferi* antigens. However, it is now known that many of those bands represented more than one protein and were cross-reactive. Using the STTT, the presence of two out of three protein bands (23, 39, 41 kilodalton) to which IgM antibody reacts, is considered positive. IgM blot alone should not be used to diagnose patients who are symptomatic over 6 weeks. Towards 4 weeks or later, IgG reactivity to 5 of 10 bands is considered positive (but note the caveats above and described in detail elsewhere [18,24]).

As an improvement, a modified two-tier test (MTTT) [19,25] has been approved by the FDA. It substitutes a “first-tier-like” immunoassay for the Western blot as the second step. Equal or improved sensitivity, without degradation of specificity or subjectivity, has been achieved. One caveat affecting both tests is that early antibiotics may blunt an expected antibody response and cause an apparent seronegative response. A likely explanation is that early antibiotic therapy leads to clearance of the pathogen prior to the development of a class-switched antibody response. As a result, antibody responses either do not develop, or individuals do not seroconvert from IgM to IgG. Therefore, it is important to know if a patient has received antibiotics. During the early phase of the disease, often between the third and sixth week, there is a robust IgM response. It is likely that the Western blot tests will be used less and less over the next few years, and replaced by a recombinant-based immunoassay. Despite limitations of the two-tiered serologic assays [18,24], the majority of suspected cases of Lyme disease should be borderline or positive in a patient who has not received treatment and more than a month or two has elapsed since possible infection. These tests have a significant negative predictive value toward ruling out the disease in endemic regions in such patients. Clinicians should enhance their interpretation of laboratory tests by consulting with the laboratory technical director where they send their tests.

Direct tests can measure specific and active infections in many cases [18]. They are often offered by clinical laboratories, but they are not yet FDA-approved. Finding pathogen nucleic acid, especially if circulating in blood or CSF, is strong evidence of an active infection. As a caveat, tissue bound pathogen nucleic acid may be remnant material and not necessarily be a measure of active infection. Direct Lyme PCR has a sensitivity of approximately 50–70% in a true EM lesion, and 20% in synovial fluid, from true Lyme arthritis.

The rheumatologist is in an excellent position to further research endeavors in Lyme disease and autoimmune disorders, as they can conduct careful examinations and sample acquisitions from this relatively restricted joint compartment. When a patient comes in with monoarthritis to an academic center, a rheumatologist is almost always consulted. The academic rheumatologist can then perform an arthrocentesis at bedside for synovial fluid analysis and possibly a biopsy of the synovium. In the case of Lyme disease, a previous study conducted during a 17-year period had samples of synovial fluid collected from 127 patients with Lyme arthritis who were seen in the Lyme disease clinics. The study found that *B. burgdorferi* DNA was detected in 75 of 88 patients with Lyme arthritis (85 percent), but in none of the 64 control patients. This presented evidence that PCR is a useful method for detecting *B. burgdorferi* DNA in synovial fluid from patients with Lyme arthritis. Although PCR testing of synovial fluid has not been standardized for widespread clinical use, *B. burgdorferi* DNA is detectable in synovial fluid by PCR in about 70 percent of patients with untreated Lyme arthritis [26].

As for a neurological workup, imaging is usually found to be normal in up to 75% of cases. Sometimes imaging shows an enhancement of the facial nerve, but this is non-specific. CSF should be obtained from a suspected neurologic Lyme disease subject, especially those with headache, fever, and neck stiffness or spinal pain. The rheumatologist should be prepared in advance to have an identified neurologist for referral. CSF results are likely to influence antibiotic choice, as mentioned below.

In consideration of other diseases in the differential, CSF studies should include cell count and differential, protein and glucose concentrations, and Gram stain and bacterial cultures. Intrathecal Lyme antibody testing for CSF serum indices should be mandatory, and checked routinely in anyone who has CSF examined for possible neurologic Lyme disease [27]. Syphilis testing can be obtained as well. Viral studies and cultures should be obtained, with testing for herpes simplex virus. Patients presenting with Lyme meningitis typically have a modest CSF pleocytosis of up to several hundred mononuclear cells per microL; the median count in acute neurologic Lyme disease is approximately 160 cells/microL (160 × 10^6^ cells/L). The CSF protein concentration is usually moderately elevated. The CSF glucose concentration is generally normal. In North American cases, a bland CSF picture may be common using traditional CSF tests [28].

## 4. Treatment of Neurologic Lyme Disease: Consideration of CNS-Penetrating Antibiotics

A full discussion of this topic is beyond the intended scope of the article. Published guidelines [29,30] cite that patients with CNS disease are likely to benefit from known CNS-penetrating antibiotics, such as intravenous therapy with ceftriaxone for 14–21 days. The rheumatologist with limited experience in treating and following patients with neurologic Lyme disease is encouraged to confer with a neurologist or an infectious disease physician experienced with neurologic Lyme disease. With appropriate antibiotic therapy for early Lyme disease, persisting neurologic sequelae have been minimized. Nevertheless, 10% or more of early treated patients may not return to their baseline. We call attention to patients with CNS involvement, and the need to discriminate between recommendations from the literature based on European patients and those for North American patients, as the disease may be different. Neurological involvement involves discussions on using oral medications, such as doxycycline and amoxicillin, for some forms of systemic or neurologic Lyme disease. Steere et al. [31] noted that in treating Lyme arthritis without neurologic symptoms at the onset, 1/18 (5.5%) patients treated with oral doxycycline and 4/16 (25%) patients treated with oral amoxicillin, later developed neurologic Lyme disease, despite resolution of the arthritis. This suggests that the nervous system was infected early and that the oral medication was ineffective against the neurologic seeding [31]. In treating systemic illness when meningitis is involved, intravenous ceftriaxone is recommended over oral doxycycline [30].

When treated early, neurologic Lyme disease has a favorable prognosis. However, it can be difficult to determine the efficacy of antibiotic therapy during treatment, as improvement may occur over weeks to months, particularly in late stage infection. In patients with post-treatment Lyme disease with persistent symptoms, long-term antibiotics over weeks to months have not been shown to yield sustained resolution [32,33,34].

## 5. Conclusions

For a multitude of reasons, patients with possible Lyme disease may present themselves to the rheumatologist. The rheumatologist is in an excellent position to evaluate patients, especially those who may have been missed at the earliest stage of Lyme disease and present with rheumatologic and/or neurologic symptoms. Accompanying suggestive neurologic symptoms should raise the possibility of neurologic Lyme disease, with further assessments. It can be difficult to diagnose a patient with neurologic Lyme disease. Therefore, it is important for a rheumatologist to initially gather a carefully elicited history from the patient.

Preparedness can maximize favorable outcomes for the patients. This includes having a go-to experienced neurologist for prompt referral and interdisciplinary management. A relationship with the laboratory servicing patients is also important to determine the best test and know when to use that test when considering neurologic Lyme disease. When examining a patient with possible endemic area exposure to *B. burgdorferi* with rheumatologic complaints, it is important to consider Lyme disease and particularly neurologic Lyme disease as a possible diagnosis.

Current knowledge and the landscape of Lyme disease are changing. Factors that favor exposure to ticks (encroachment of residences near wooded areas and climate change) may play a role in future to enhance the incidence of Lyme and tick-borne diseases [35].

Key guiding points in suspected Lyme disease are as follows: arthralgias are far more common than arthritis, neurologic manifestations are far more frequent than arthritis, EM in true cases of Lyme disease most often has a non-classic appearance more frequently than a classic bull’s-eye lesion, and even the classic appearing EM is not totally pathognomonic because of mimicking lesions. Because neurologic involvement in Lyme disease is so common, recognition and timely treatment should be encouraged.

## Figures and Tables

**Table 1:** Rheumatologic features of Lyme disease.

Lyme Disease Stage	Rheumatologic Syndromes
Early Localized (Erythema migrans)	Migratory arthralgias and myalgias
Early Disseminated	Migratory bursitis (rare), tendonitis (rare), myalgias, arthralgias
Late	Intermittent or persistent mono- or oligoarthritis of the large joints (most commonly knee; less common shoulder, ankle, elbow, temporomandibular joint, wrist) Absence of small joint involvement Asymmetric involvement Tenderness on joint palpation Joint effusions Lasts weeks to months

**Table 2:** Common factors that raise concern for neurologic Lyme disease *.

Demographics	Endemic area exposure Seasonality (late spring–early fall) Prior Lyme disease in subject, or their immediate family Ixodid tick exposure
Symptoms	Headache Cognitive Issues Fatigue Spine pain (neck, mid/lower back pain) Numbness/Paresthesias
Signs	Unilateral or bilateral facial weakness (especially with tearing, hyperacusis, taste issues) Meningismus (stiff neck) Radicular sensory and motor deficits

* Less common features are related to aspects of transverse myelitis [12], cerebrovascular events [13], and vasculitis [14,15] all of which are likely to come to the more direct attention of a neurologist or dermatologist rather than a rheumatologist.

## Data Availability

Not applicable.
